# *Dendrobium officinale* Orchid Extract Prevents Ovariectomy-Induced Osteoporosis *in Vivo* and Inhibits RANKL-Induced Osteoclast Differentiation *in Vitro*

**DOI:** 10.3389/fphar.2017.00966

**Published:** 2018-01-15

**Authors:** Qi Wang, Cheng-Ting Zi, Jing Wang, Yu-Na Wang, Ye-Wei Huang, Xue-Qi Fu, Xuan-Jun Wang, Jun Sheng

**Affiliations:** ^1^Key Laboratory of Pu-erh Tea Science, Ministry of Education, Yunnan Agricultural University, Kunming, China; ^2^Tea Research Center of Yunnan, Kunming, China; ^3^College of Tea Science, Yunnan Agricultural University, Kunming, China; ^4^College of Food Science and Technology, Yunnan Agricultural University, Kunming, China; ^5^College of Life Sciences, Jilin University, Changchun, China; ^6^State Key Laboratory for Conservation and Utilization of Bio-Resources in Yunnan, Kunming, China

**Keywords:** DOE, postmenopausal osteoporosis, ovariectomy, bone quality, osteoclastogenesis

## Abstract

**Background:** *Dendrobium officinale*, a traditional Chinese medical herb with high value that is widely used in Asia, possesses many positive effects on human health, including anti-chronic inflammation, anti-obesity, and immune modulation properties; however, whether *D. officinale* has inhibitory effects on postmenopausal osteoporosis remains unknown.

**Objective:** We investigated the effects of *D. officinale* extract (DOE) on ovariectomy-induced bone loss *in vivo* and on osteoclastogenesis *in vitro*.

**Methods:** *In vivo*, female rats were divided into a sham-operated (sham) group and five ovariectomized (OVX) subgroups: OVX with vehicle (OVX), OVX with Xian-Ling-Gu-Bao capsule (240 mg/kg body weight/day), and OVX with low-, medium-, and high-dose DOE (150, 300, and 600 mg/kg body weight/day, respectively). Animals in each group were administered their corresponding treatments for 13 weeks. Body weight, serum biochemical parameters, uterine and femoral physical parameters, bone mineral density (BMD), bone biomechanical properties, and bone microarchitecture were obtained. *In vitro*, the effects of DOE on osteoclastogenesis were examined using RAW264.7 cells. The effects of DOE on osteoclastogenesis and the expression of osteoclast-specific marker genes and proteins were determined.

**Results:** DOE effectively ameliorated serum biochemical parameters, especially alleviated estradiol (E2) deficiency and maintained calcium and phosphorus homeostasis. DOE improved uterine and femoral physical parameters. In addition, DOE improved femoral BMD and biomechanical properties. DOE significantly ameliorated bone microarchitecture. Moreover, DOE inhibited osteoclastogenesis independent of its cytoxicity and suppressed the expression of osteoclast-specific marker genes and proteins.

**Conclusion:** DOE can effectively prevent ovariectomy-induced bone loss *in vivo* and inhibit osteoclastogenesis *in vitro*.

## Introduction

Bone is dynamically molded, shaped, and repaired throughout life via coordination between bone-forming osteoblasts and bone-resorping osteoclasts (Boyle et al., 2003; Xu et al., 2017). Thus, imbalances in bone remodeling can result in various osteopathic diseases, especially osteoporosis which is caused by excess osteoclast activity and includes rheumatoid arthritis, periodontal disease, multiple myelomas, and metastatic cancers, as well as osteoporosis (Boyle et al., 2003; Wang C. et al., 2017). This disease is a common, chronic metabolic condition associated with decreased bone mass and microarchitectural deterioration; these changes lead to increased bone fragility that predisposes affected individuals to an increased risk of fractures, particularly postmenopausal women (Yin et al., 2004; Baek et al., 2016; Curtis et al., 2016; Liu et al., 2016).

Postmenopausal osteoporosis (PMOP) is the most common primary form of this disease; morbidity as a result of this condition has been increasing rapidly because of changing demographics and increasing life expectancies (Ye et al., 2015; Chen et al., 2017). Numerous studies have demonstrated that estrogen deficiency is closely associated with PMOP pathogenesis (Ye et al., 2015; Chen et al., 2017; Liu et al., 2017); this is an important observation because a deficiency in this hormone is common in women after menopause (Liu et al., 2017), and it is well-established that estrogen plays a vital role in maintaining bone homeostasis and regulating remodeling (Chen et al., 2017; Streicher et al., 2017). It is also clear that estrogen has a number of osteoprotective effects; on the one hand, this hormone inhibits receptor activator expression of the nuclear factor-κB ligand (RANKL), a cytokine essential for osteoclast bone resorption, and therefore suppresses the differentiation and activation of these cells. Estrogen also directly stimulates osteoblasts to secrete osteoprotegerin, a cytokine antagonist for RANKL that blocks osteoclastogenesis and bone resorption (Boyle et al., 2003; Li C. et al., 2011; Chen et al., 2017; Streicher et al., 2017). Enhanced osteoclastogenesis contributes to the development of PMOP; thus, inhibiting the differentiation and activation of osteoclasts is a critical management strategy for this disease.

A number of therapeutic agents are currently used to treat postmenopausal women at risk of developing osteoporosis, including hormone replacement therapy (Curtis et al., 2016). Although anti-osteoporotic drugs can be used to elevate bone mineral density (BMD) and reduce the risk of fractures, recent evidence suggests that their long-term use may have adverse effects on human health (Curtis et al., 2016). It is therefore urgent to develop alternative agents with less side effects for the prevention and treatment of osteoporosis.

It is encouraging that natural products from medicinal plants have been shown to be valuable sources for the development of novel drugs against a range of diseases (Niu et al., 2012; Sacco et al., 2013). One example is the orchid *Dendrobium officinale* (Zhao et al., 2014), a traditional Chinese high-value medicinal herb that is widely used across Asia (Zhao et al., 2014; Sun et al., 2016). This plant contains polysaccharides, stillnoids, alkaloids, amino acids, and trace elements and is known to exert a number of positive effects on human health (Li et al., 2010; Li L. et al., 2011), including anti-tumor, anti-oxidative, anti-chronic inflammation, anti-obesity, anti-hypertensive, hypoglycemic, neuron protection, and immune system modulation activities (Xiao et al., 2011; Zhao et al., 2014; Sun et al., 2016; Lee et al., 2017; Wang Q. et al., 2017). Because of these known positive effects on human health, we hypothesize that *D. officinale* extract (DOE) will also exhibit anti-osteoporotic properties. The aim of this study is to systematically investigate whether, or not, DOE exerts anti-osteoporotic effects on *in vivo* ovariectomy-induced osteoporosis. We also evaluate the effects of this extract on *in vitro* RANKL-induced osteoclast differentiation.

## Materials and Methods

### Plant Materials and Water Extract Preparation

The stems of *D. officinale* were collected in Yunnan Province in 2011 and identified by Frof. Kaicong Fu of Peru’s National Institute of Tradition Medicine. A voucher specimen (2011-DPE-1) was deposited in the Key Laboratory of Pu-erh Tea Science of Yunnan Agricultural University. The stems of *D. officinale* (134.8 g) were extracted three times with 1000 ml of water each time (30 min) under refluxed. The extract was decanted, filtered, and vacuum-dried to obtain the crude water extract 17.25 g.

### Total Polysaccharides Determination

Total polysaccharides were quantified by the phenol–sulfuric acid colorimetric methods (Dubois et al., 1980), using D-glucose as the standard. Briefly, 1.0 ml of the properly diluted sample was mixed with 1.0 ml deionized water in 10.0 ml test tube with stopper. Then, 1.0 ml phenol (5%, w/v) was added, followed by the addition of 5.0 ml H_2_SO_4_. The solution was mixed and heated in a boiling water bath for 20 min, removed, cooled 5 min in an ice bath, and the absorbance was measured at 488 nm. A standard solution of D-glucose was used to prepare a calibration curve. The results were expressed as milligram D-glucose per gram of dry extract.

### Total Phenolics Determination

The total phenolic content was determined according to the Folin–Ciocalteau method (Singleton and Rossi, 1995; Gursoy et al., 2009), using gallic acid as the standard. One milliliter of the properly diluted sample was mixed with 0.3 ml Folin–Ciocalteau reagent in a 10.0 ml volumetric flask. Then, 1.5 ml Na_2_CO_3_ (7%, w/v) was added and incubated at room temperature for 20 min, and the absorbance was measured at 760 nm. The results were calculated and expressed as milligram of gallic acid per gram of dry extract.

### Total Flavonoids Determination

The total flavonoid content was determined by a colorimetric assay according to previous described (Siddhuraju and Becker, 2003). One milliliter of the properly diluted sample was mixed with 4.0 ml of EtOH (50%, v/v) in a 10.0 ml volumetric flask, then, 0.3 ml of NaNO_2_ (5%, w/v) was added. Six minutes later, 0.3 ml of Al(NO_3_)_3_ (10%, w/v) was added. After incubation for 6 min, 4.0 ml of NaOH (1.0 M) was added to the mixture, followed by the addition of 0.4 ml of EtOH (50%, v/v). The mixture solution was mixed and incubated 15 min, and the absorbance at 510 nm was measured against a blank. A standard solution of rutin was used to prepare a calibration curve. The results were expressed as milligram of rutin per gram of dry extract.

### High Performance Liquid Chromatography (HPLC) Analysis

The crude water extract of *D. officinale* (80.0 mg) was dissolved with hot water (20 ml), then, added absolute ethyl alcohol (50 ml) and subsided in 4°C freezer overnight. The precipitate was filtrated, and the filtrate was concentrated. The residue (22.8 mg) was mixed with 1.0 ml CH_3_OH in a 2.0 ml volumetric flask, then, 1.0 ml of CH_3_OH was added. All sample solutions were filtered through a 0.45-μm MFS membrane before injection into the HPLC system. Analytical HPLC was performed on an Agilent 1260 liquid chromatograph equipped with a ZORBAX SB-C18 (4.6 × 250 mm, 5 μm) column. The optical mobile phase for the analysis was gradient elution system consisting of solvent A (water, 0.1% acetic acid) and solvent B (methyl alcohol). The gradient program was as following: 0–5 min, 25% solvent B; 5–10 min, 25–30% solvent B; 10–25 min, 30–40% solvent B; 25–45 min, 40–55% solvent B; 45–60 min, 55–70% solvent B. The detection wavelength was 280 nm. The injection volume was 10 μl. The flow rate was 1.0 ml/min, the column temperature was set 30°C.

### Animals

Twelve-week-old female Wistar rats (Cat. No. SCXK-(Ji)2008-0005) were obtained from the Laboratory Animal Center of Jilin University (Jilin, China). These rats were given access to normal rodent chow and water *ad libitum* and kept under a 12 h dark, 12 h light cycle for 1 week to allow them to adapt to their housing environment prior to the experiment. All animals were maintained in a room equipped with an air-filter system, while their cages and water were sterilized and replaced once daily. This study was carried out in accordance with the recommendations of the Institutional Animal Care and Use Committee of Yunnan Agricultural University (Yunnan, China). The protocol was approved by the Animal Experiments Ethics Committee of Yunnan Agricultural University.

### Groups and Treatments

Subsequent to acclimatization for 2 week, rats were either sham operated (sham group, *n* = 10) or bilaterally ovariectomized (OVX subgroup, *n* = 50) as previously described (Liu et al., 2017). Thus, following a 2-week recovery period, OVX rats were randomly subdivided into 5 groups of 10, then were given distilled water (OVX group), a Xian-Ling-Gu-Bao (XLGB) capsule (240 mg/kg body weight/day; Guizhou Tongjitang Pharmaceutical, Guizhou, China) (positive XLGB group) (Dai et al., 2013; Gao et al., 2013; Liu et al., 2017), 150 mg/kg body weight/day DOE (low treatment group), 300 mg/kg body weight/day DOE (medium treatment group), or 600 mg/kg body weight/day DOE (high treatment group) by oral gavage. XLGB capsules are common anti-osteoporosis drugs in China and have been used in clinic (Dai et al., 2013; Gao et al., 2013). The dosage of XLGB capsules in this study was determined and calculated according to prior studies (Liu et al., 2017). Animals in each group were administered their respective treatments for 13 weeks, and individual body weights were recorded weekly. Blood samples for serum and plasma isolation were collected after the last treatment administration from the abdominal artery of rats that had fasted overnight and then been anesthetized. Fresh femora from both sides as well as the uterus of each animal were then dissected and weighed. All samples were maintained in 10% neutral formaldehyde for subsequent histological analysis.

### Serum Biochemical Parameter Analysis

Blood samples were allowed to clot at room temperature before being centrifuged at 3,000 × *g* for 5 min at 4°C. Serum was then collected and stored at -80°C prior to analysis. The serum concentrations of total cholesterol (TC), triglyceride (TG), high-density lipoprotein-cholesterol (HDL-C), low-density lipoprotein-cholesterol (LDL-C), glucose, and alkaline phosphatase (ALP) were all measured using relevant assay kits (Shenzhen Mindray Bio-Medical Electronics, Shenzhen, China). The calcium and phosphorus levels were also measured (Zhongsheng Beikong Bio-Technology and Science, Beijing, China). The serum estradiol (E2) and bone Gla protein (BGP) levels were, respectively, determined using radioimmunoassay kits from Shenzhen Lalwen Bioengineering Technology (Shenzhen, China) and Beijing North Institute of Biological Technology (Beijing, China). The acid phosphatase (ACP) level was analyzed with kit from Nanjing Jiancheng Bioengineering Institute (Nanjing, China). All kit procedures were followed by the manufacturer’s instructions.

### Determination of Organ Coefficients, Femoral Diameter, and Length

Femur and uterus organ coefficients were calculated as followed: organ coefficient of the femur = weight of the femur/body weight; organ coefficient of the uterus = wet weight of the uterus/body weight (Liu et al., 2017), while femoral diameter and length were measured directly.

### BMD and Biomechanical Assays

The BMD and biomechanical properties (i.e., maximum deflection and maximum load) of the left femur were measured as described previously (Liu et al., 2017), enabling calculation of femoral BMD, maximum deflection, and maximum load parameters. Briefly, BMD was automatically measured using dual-energy X-ray absorptiometry (DEXA) and biomechanical properties were evaluated by the three-point bending flexural test method (Gao et al., 2013).

### Histological Analysis

Histological analyses were performed on the uterus and right femur, again as previously described (Liu et al., 2017). Briefly, the fresh tissues of rat right femur were instantly collected, fixed in 10% neutral formaldehyde for 72 h, and removed and dehydrated. And fixed tissue samples were embedded in paraffin, cut into 4 μm sections using a slicer (Leica, Germany), and then stained with hematoxylin and eosin (H&E) and observed under a CKX41 microscope (Olympus, Tokyo, Japan). Images of cortical and trabecular bone were obtained by a BI-2000 Medical Image Analysis System (Chengdu Technology and Market Co., Ltd., Chengdu, China) and endometrial height, cortical bone thickness, and trabecular bone area were measured by a professional software.

### Cell Cultures

RAW264.7 murine macrophages were purchased from the American Type Culture Collection (Manassas, VA, United States) and were maintained in DMEM high glucose medium (Thermo Fisher Scientific, Pittsburgh, PA, United States) supplemented with 10% fetal bovine serum (HyClone, San Francisco, CA, United States), and 1% penicillin/streptomycin (Solarbio, Beijing, China) at 37°C in a humidified atmosphere containing 95% air and 5% CO_2_.

### Cell Viability Assay

RAW264.7 cells (3 × 10^4^ cells/well) were then cultured with, and without, DOE (between 5 and 160 μg/ml) for either 24 or 48 h in 96-well plates. Cell viability was then determined following drug treatment using the standard 3-(4,5-dimethylthiazol-2-yl)-2,5-diphenyltetrazolium bromide (MTT; Sigma–Aldrich, St. Louis, MO, United States) method as described previously (Liu et al., 2017). Absorbance at 492 nm was measured using a microplate reader (Thermo Fisher Scientific, Pittsburgh, PA, United States), and proliferation was normalized against control cells.

### *In Vitro* Osteoclastogenesis Assay

RAW264.7 cells (2 × 10^3^ cells/well) were then cultured with, and without, RANKL (50 ng/ml; R&D Systems, Minneapolis, MN, United States) in the presence of XLGB (10 μg/ml) or DOE (either 40 or 80 μg/ml) in 96-well plates. Following incubation for 5 days, a Tartrate-Resistant Acid Phosphatase (TRAP) Staining Kit (Sigma–Aldrich, St. Louis, MO, United States) was used to identify osteoclasts, following the manufacturer’s instructions. Images were acquired using a digital camera attached to the microscope and the number of mature osteoclasts containing more than three nuclei was calculated, again as described previously (Jiang et al., 2014; Wang C. et al., 2017).

### Quantitative Real-Time PCR (qRT-PCR) Analysis

RAW264.7 cells (1.2 × 10^5^ cells/well) were cultured with, and without, RANKL (50 ng/ml) in the presence of either XLGB (10 μg/ml) or DOE (40, 80 μg/ml) in 96-well plates for 48 h. Total RNA was then extracted from cultured cells using the TransZol Up Reagent (TransGen Biotech, Beijing, China), and gene expression was detected via quantitative real-time PCR (qRT-PCR) analysis which was performed using SYBR Premix Ex TaqTM II (Tli RNaseH Plus, TaKaRa Bio), as previously described (Liu et al., 2017). The primers sequences (Generay Biotech, Shanghai, China) are shown in the **Table 1**.

**Table 1:** The qRT-PCR primers used in this study.

Genes	Forward (5′–3′)	Reverse (5′–3′)
*GADPH*	AACTTTGGCATTGTGGAAGG	ACACATTGGGGGTAGGAACA
*TRAP*	GCTGGAAACCATGATCACCT	GAGTTGCCACACAGCATCAC
*c-Fos*	CAAGCGGAGACAGATCAAC	TTCCTTCTCTTTCAGCAGAT
	TTG	TGG
*c-Src*	CCAGGCTGAGGAGTGGTACT	CAGCTTGCGGATCTTGTAGT
*β3-Integrin*	TGACATCGAGCAGGTGAAAG	GAGTAGCAAGGCCAATGAGC
*Cathepsin K*	CTTCCAATACGTGCAGCAGA	TCTTCAGGGCTTTCTCGTTC
*NFATc1*	TGGAGAAGCAGAGCACAGAC	GCGGAAAGGTGGTATCTCAA

### Western Blot Analysis

The use of western blot analysis has been described previously (Liu et al., 2017). In this study, RAW264.7 cells (4 × 10^5^ cells/well) were cultured with, and without, RANKL (50 ng/ml) in the presence of XLGB (10 μg/ml) or DOE (40, 80 μg/ml) in 60 mm plates for 48 h. Protein extracts were then prepared using the RIPA buffer (Solarbio, Beijing, China), separated using SDS–PAGE gels, transferred to PVDF membranes (Millipore, Darmstadt, Germany), and subject to immunoblotting using primary antibodies against NFATc1 (7A6), c-Src (17AT28), cathepsin K (E-7), and c-Fos (C-10) (Santa Cruz, CA, United States), as well as an antibody against β-tubulin (CST, Framingham, MA, United States).

### Statistical Analysis

All values reported here are presented as means ± the standard error of the mean (SEM) from three or more independent replicates. Data were analyzed using the one-way ANOVA and Tukey’s test for independent groups; *P* < 0.05 was considered statistically significant and representative images presented.

## Results

### Contents of Total Polysaccharides, Total Phenolics, Total Flavonoids, and Chemical Composition Analysis

**FIGURE 1:**
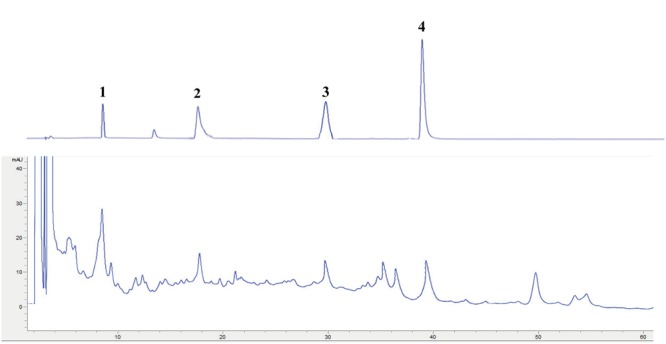
HPLC chromatogram of reference characteristic of compounds: Vicenin 2 (1); Narigenin (2); Daucosterol (3); and β-Sitosterol (4).

Polysaccharides are reported as the major active constituents of *Dendrobium* species, and have attracted more attention with multiple pharmacological activities, such as anti-oxidant, anti-aging, anti-tumor, anti-mutagenic, and immunoregulation effects (Inubushi et al., 1966; Tian et al., 2013; Chen et al., 2014; He et al., 2015; Jiao et al., 2015). The total polysaccharides contents in the water extract of *D. officinale* were determined in this work, and the values of total polysaccharides were expressed in terms of milligrams of D-glucose per gram dry extract. The result showed that total polysaccharides values: 5.5 ± 0.1 mg D-glucose/g dry extract, the reference (Li et al., 2012) also showed a similar result. Phenolics have attracted considerable attention because of their various biological activities, including anti-oxidant, anti-tumor, anti-atherogenic, and cardioprotective effects (Tai et al., 2011). The total phenolics contents in the water extract were determined in this research, and were expressed in terms of milligrams of gallic acid per gram of dry extract. The result showed that total phenolics values: 11.4 ± 1.2 mg gallic acid/g dry extract. Flavonoids are also known to be contributors to the anti-oxidant, anti-tumor, and anti-atherogenic capacities (Teixeira et al., 2005). The total flavonoids content of the water extract of *D. officinale* were determined and expressed in terms of milligram of rutin per gram dry extract by comparison with standard rutin treated in the same conditions. The result showed that total flavonoids values: 17.1 ± 1.6 mg rutin/g dry extract. Compared with the previous studies (Inubushi et al., 1966; Tian et al., 2013; Chen et al., 2014; Li Y. et al., 2014; He et al., 2015; Jiao et al., 2015; Yang et al., 2015), polysaccharide and bibenzyl are reported as the major constituents of *D. officinale*. The major chemical composition of the water extract was analyzed by HPLC system in this study. The reference characteristic of compounds (Vicenin 2, Narigenin, Daucosterol, and β-Sitosterol) was taken as control and the results are presented in **Figure 1**. Among the chromatographic peaks, most of them have been identified.

### DOE Effects on OVX-Induced Weight Gain in Rats

We used a model of OVX-induced osteoporosis in rats in order to investigate the effects of DOE on PMOP *in vivo*. Body weight in each group was gradually increased over the course of this experiment (**Table 2**); thus, the rats in the OVX group weighed significantly more than their sham-operated counterparts after 6 weeks (*P* < 0.05, *P* < 0.01, and *P* < 0.001). Compared with the OVX group, results show that OVX-induced body weight gains were not affected by either XLGB or DOE treatments.

**Table 2:** Effects of DOE on body weight (g) in OVX rats.^#^

Time (week)	Sham	OVX	XLGB	Low-dose	Medium-dose	High-dose
0	267.3 ± 19.6	266.9 ± 33.3^A^	268.9 ± 38.2^a^	266.3 ± 54.9^a^	267.4 ± 41.0^a^	266.8 ± 38.4^a^
1	267.1 ± 19.2	282.1 ± 37.7^A^	278.2 ± 42.1^a^	285.2 ± 52.4^a^	282.1 ± 43.2^a^	271.2 ± 35.7^a^
2	272.5 ± 21.1	283.3 ± 36.4^A^	284.8 ± 34.9^a^	296.6 ± 52.5^a^	293.4 ± 37.9^a^	283.6 ± 37.2^a^
3	277.1 ± 20.6	289.2 ± 34.1^A^	290.0 ± 34.1^a^	306.9 ± 55.4^a^	298.3 ± 40.8^a^	295.1 ± 40.1^a^
4	282.7 ± 21.8	301.4 ± 32.5^A^	303.3 ± 38.2^a^	320.3 ± 57.9^a^	310.21 ± 43.8^a^	304.5 ± 45.3^a^
5	289.1 ± 21.8	311.5 ± 32.5^A^	315.3 ± 41.4^a^	329.4 ± 61.2^a^	325.7 ± 43.6^a^	314.9 ± 53.6^a^
6	290.5 ± 21.7	321.5 ± 30.8^C^	325.0 ± 44.2^a^	339.3 ± 67.7^a^	336.8 ± 46.9^a^	324.6 ± 59.6^a^
7	305.2 ± 27.6	331.4 ± 30.5^B^	334.1 ± 48.0^a^	346.1 ± 68.0^a^	347.7 ± 47.6^a^	332.7 ± 60.3^a^
8	289.5 ± 24.8	336.7 ± 25.2^D^	336.7 ± 48.8^a^	347.9 ± 72.4^a^	351.9 ± 47.6^a^	337.7 ± 58.7^a^
9	299.1 ± 30.3	345.3 ± 26.5^D^	343.3 ± 52.1^a^	358.3 ± 73.4^a^	362.3 ± 50.9^a^	345.8 ± 59.4^a^
10	295.7 ± 31.8	353.3 ± 28.0^D^	340.0 ± 558^a^	361.7 ± 80.2^a^	368.0 ± 52.7^a^	350.9 ± 62.1^a^
11	296.4 ± 29.6	354.4 ± 29.1^D^	345.0 ± 57.4^a^	366.0 ± 83.6^a^	372.5 ± 53.7^a^	352.4 ± 62.2^a^
12	301.8 ± 27.3	357.2 ± 32.4^D^	350.9 ± 58.6^a^	372.8 ± 86.6^a^	374.5 ± 57.0^a^	335.1 ± 67.4^a^
13	303.3 ± 27.4	360.9 ± 32.5^D^	353.1 ± 60.0^a^	377.2 ± 90.0^a^	377.7 ± 59.7^a^	363.5 ± 68.0^a^

*^#^ Data are presented as means ± SEM (*n* = 10). ^A^*P* > 0.05, ^B^*P* < 0.05, ^C^*P* < 0.01, and ^D^*P* < 0.001, OVX group compared with Sham at the same time; ^a^*P* > 0.05, XLGB and DOE group compared with OVX group at the same time.*

### DOE Treatment Ameliorates Serum Biochemical Parameters in OVX Rats

**FIGURE 2:**
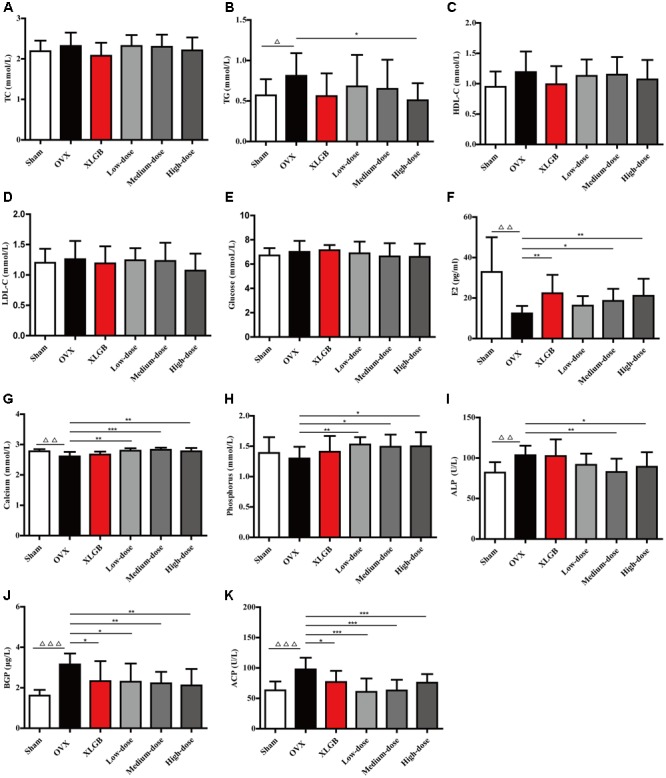
DOE treatment ameliorates serum biochemical parameters in OVX rats: **(A)** TC; **(B)** TG; **(C)** HDL-C; **(D)** LDL-C; **(E)** Glucose; **(F)** E2; **(G)** Calcium; **(H)** Phosphorus; **(I)** ALP; **(J)** BGP; and **(K)** ACP concentrations. All data are presented as means ± SEM (*n* = 10). ^∆^*P* < 0.05, ^∆∆^*P* < 0.01, and ^∆∆∆^*P* < 0.001 versus the sham group, and ^∗^*P* < 0.05, ^∗∗^*P* < 0.01, and ^∗∗∗^*P* < 0.001 versus the OVX group.

Although treatment with DOE did not mitigate body weight gain in OVX rats, we nevertheless determined whether, or not, this extract could enhance serum biochemical parameters. Results show no significant differences in serum TC concentration in each group (**Figure 2A**). The TG level in the OVX group was significantly increased in comparison with the sham group (*P* < 0.05; **Figure 2B**). Interestingly, treatment with both XLGB and DOE decreased the TG level in OVX rats compared with the OVX group, while a high-dose DOE treatment was most significant (*P* < 0.05). As no significant differences were found in HDL-C, LDL-C, and glucose concentrations in each group (**Figures 2C–E**) these results suggest that DOE treatment improved the lipid and glucose metabolisms in OVX rats to a certain degree.

We then detected serum biomarkers, including E_2_, calcium, phosphorus, ALP, BGP, and ACP to further explore the effects of DOE on bone metabolism in OVX rats (**Figures 2F–K**). The results of these comparisons show that the E_2_ level in the OVX group decreased significantly compared with the sham group (*P* < 0.01; **Figure 2F**). Thus, as expected, XLGB treatment significantly increased the E_2_ level compared with the OVX group (*P* < 0.01). Similarly, the extent of deficiency in this compound was alleviated by treatment with DOE in medium- and high-doses exerting significant effects (*P* < 0.05 or *P* < 0.01).

Results show that both the calcium and phosphorus levels in the OVX group were decreased compared with the sham group, with a significant difference observed in the level of the former (*P* < 0.01; **Figures 2G,H**). OVX rats treated with XLGB also experienced a significant increase in calcium and phosphorus levels compared to the OVX group, while DOE treatment significantly increased the serum levels of both these elements (*P* < 0.05, *P* < 0.01, or *P* < 0.001). This result indicates that DOE can effectively maintain the homeostasis of these two elements in OVX rats.

Results also show that bone formation biomarkers, including ALP and BGP, significantly increased in the OVX group compared to the sham group (*P* < 0.01 or *P* < 0.001; **Figures 2I,J**). Interestingly, DOE treatment at medium- and high-doses significantly decreased the ALP level compared with the OVX group (*P* < 0.05 or *P* < 0.01), while both XLGB and DOE treatments significantly decreased the BGP level in OVX rats (*P* < 0.05 or *P* < 0.01). Concentration of the bone resorption biomarker ACP was significantly increased in the OVX group compared to the sham group (*P* < 0.001; **Figure 2K**), while the level of this marker significantly decreased following treatments with XLGB and DOE (*P* < 0.05 or *P* < 0.001). These results collectively demonstrated that DOE treatment effectively ameliorates serum biochemical parameters in OVX rats.

### DOE Treatment Improves Uterine and Femoral Physical Parameters in OVX Rats

**FIGURE 3:**
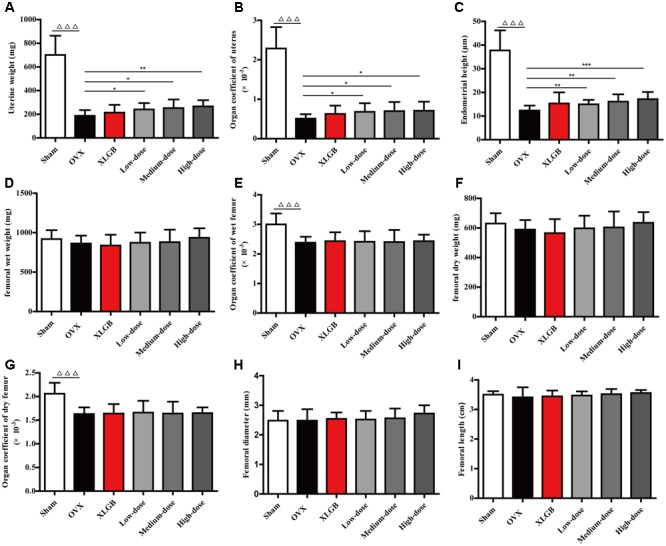
DOE treatment improves uterine and femoral physical parameters in OVX rats: **(A)** uterine weight; **(B)** organ coefficient; **(C)** endometrial height; **(D)** femoral wet weight; **(E)** organ coefficient; **(F)** femoral dry weight; **(G)** organ coefficients; **(H)** femoral diameter; and **(I)** femoral length. All data are presented as means ± SEM (*n* = 10). ^∆∆∆^*P* < 0.001 versus the sham group, and ^∗^*P* < 0.05, ^∗∗^*P* < 0.01, and ^∗∗∗^*P* < 0.001 versus the OVX group.

Uterine and femoral physical parameters in OVX rats were measured to further examine whether DOE treatment protects against the damage induced by ovariectomy (**Figure 3**). Results show that uterine weight, the organ coefficient of the uterus, and endometrial height in the OVX group all significantly decreased compared with the sham group (*P* < 0.001; **Figures 3A–C**); notably, treatment with DOE significantly enhanced these parameters compared with the OVX group (*P* < 0.05, *P* < 0.01, or *P* < 0.001). The wet and dry weight of femora was almost indistinguishable between all the groups (**Figures 3D–F**). Compared with the sham group, organ coefficients of wet and dry femora in the OVX group significantly decreased (*P* < 0.001; **Figures 3E–G**), while XLGB and DOE treatments appeared to increase these organ indices compared with the OVX group even though significant differences were not observed. Results show no significant differences in femoral diameter and length between groups, although XLGB and DOE treatments appeared to enhance these variables compared with the OVX group. These data collectively indicate that DOE treatment improves uterine and femoral physical parameters in OVX rats.

### DOE Treatment Improves Bone Quality in OVX Rats

**FIGURE 4:**
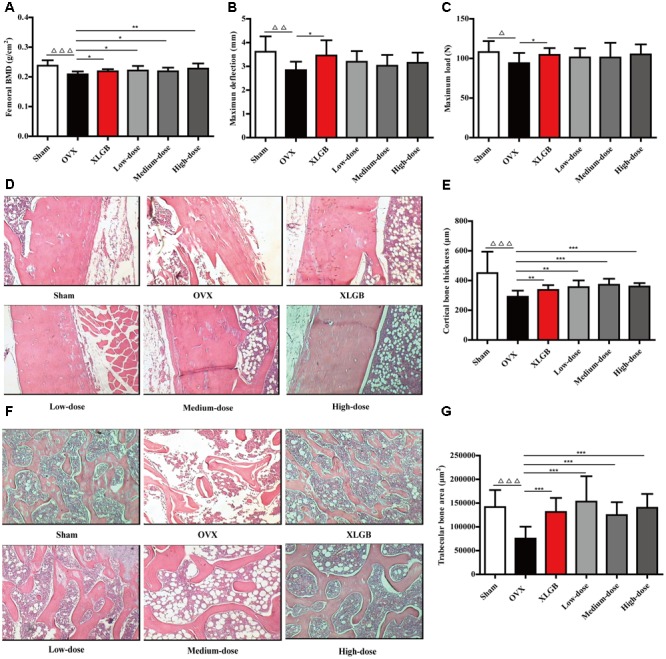
DOE treatment improves bone quality in OVX rats: **(A)** femoral BMD; **(B)** maximum deflection; **(C)** maximum load; **(D)** cortical bone tissue stained with H&E; **(E)** calculated cortical bone thickness; **(F)** trabecular bone tissue stained with H&E; and **(G)** calculated trabecular bone area. Representative images were acquired using a medical image analysis system at an original magnification of ×400. All data are presented as means ± SEM (*n* = 10). ^∆^*P* < 0.05, ^∆∆^*P* < 0.01, and ^∆∆∆^*P* < 0.001 versus the sham group, and ^∗^*P* < 0.05, ^∗∗^*P* < 0.01, and ^∗∗∗^*P* < 0.001 versus the OVX group.

Measures of BMD, biomechanical properties, and microarchitecture have all been widely applied to evaluate bone quality in osteoporosis models (Kuo and Chen, 2017; Liu et al., 2017). Thus, to further investigate whether DOE treatment improved bone quality in OVX rats, we calculated the evaluation indexes outlined above (**Figure 4**). Comparisons with the sham group show that femoral BMD in the OVX group decreased significantly (*P* < 0.001; **Figure 4A**), while compared with the OVX group, OVX-induced femoral BMD decrease was significantly reversed by XLGB and DOE treatments (*P* < 0.05 or *P* < 0.01). Femoral biomechanical properties, including maximum deflection and load, were also significantly decreased in the OVX group compared to the sham group (*P* < 0.05 or *P* < 0.01; **Figures 4B,C**). OVX rats treated with XLGB exhibited a marked increase in femoral maximum deflection and load compared with the OVX group (*P* < 0.05), while DOE treatment enhanced these parameters compared to the OVX group, albeit not significantly.

We then directly examined and quantified femoral cortical and trabecular bone microarchitecture using H&E staining to further confirm the effects of DOE on OVX-induced osteoporosis (**Figures 4D–G**). As expected, ovariectomies caused significant reductions in both cortical bone thickness (*P* < 0.001; **Figures 4D,E**) and trabecular bone area (*P* < 0.001; **Figures 4F,G**) in the OVX group compared to the sham group, while OVX rats treated with XLGB and DOE experienced a dramatic increase in cortical bone thickness (*P* < 0.05, *P* < 0.01, or *P* < 0.001) as well as a striking increase in trabecular bone area (*P* < 0.001) compared with OVX rats treated with the distilled water. Collectively, these results clearly demonstrate that DOE treatment effectively prevents *in vivo* OVX-induced osteoporosis.

### DOE Treatment Inhibits RANKL-Induced Osteoclastogenesis

It is well-known that excess osteoclast differentiation and activation contributes to the development of osteoporosis (Boyle et al., 2003). Thus, to understand the mechanisms underlying how DOE prevents estrogen deficiency-induced osteoporosis, we investigated the effect of this extract on *in vitro* RANKL-induced osteoclastogenesis.

**FIGURE 5:**
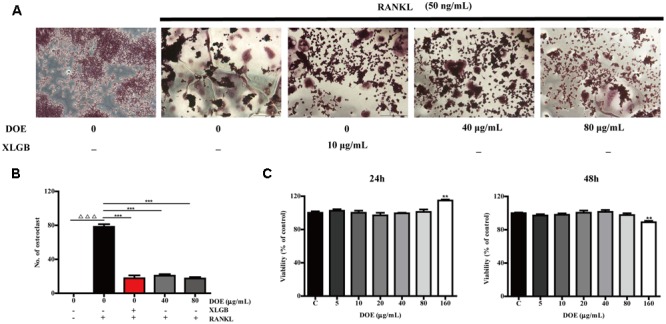
DOE treatment inhibits RANKL-induced osteoclastogenesis. **(A)** RAW264.7 cells were cultured for 5 days with RANKL (50 ng/ml) in the presence of XLGB (10 μg/ml) or the indicated concentrations of DOE and then stained for TRAP. **(B)** TRAP-positive multinucleated cells with more than five nuclei were considered mature osteoclasts, as observed under a light microscope. **(C)** The effects of DOE on the viability of RAW264.7 cells as determined by the MTT assay. Representative images were acquired using a light microscope (magnification ×200). Values are means ± SEM for three independent experiments. ^∆∆∆^*P* < 0.001 versus the control, and ^∗^*P* < 0.05, ^∗∗^*P* < 0.01, and ^∗∗∗^*P* < 0.001 versus just RANKL-treated cells.

Observations show that numerous TRAP-positive multinucleated osteoclasts formed when RAW264.7 cells were incubated with RANKL for 5 days (**Figure 5A**). At the same time, however, DOE treatment significantly inhibited RANKL-induced osteoclast formation (*P* < 0.001; **Figures 5A,B**). Thus, to eliminate the possibility that DOE suppressed osteoclastogenesis is the result of toxicity of this extract on RAW264.7 cells, we performed a MTT assay. Results show that, as expected, concentrations of up to 80 μg/ml DOE had no toxic effects on RAW264.7 cells when they were treated with the extract for 24 and 48 h, respectively (**Figure 5C**). This outcome demonstrates that the inhibitory effects of DOE on osteoclastogenesis are independent of cytoxicity.

### DOE Treatment Reduces the Expression of Osteoclast-Specific Marker Genes and Proteins

**FIGURE 6:**
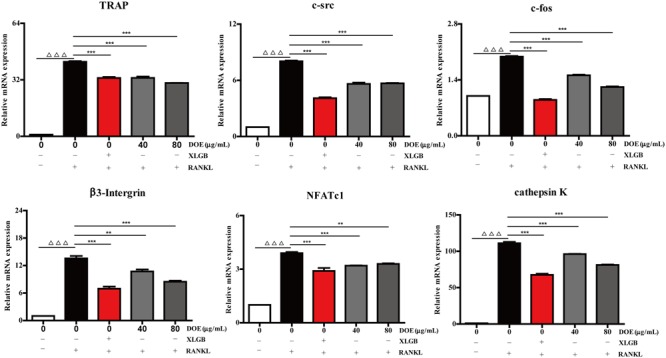
DOE treatment reduces the expression of osteoclast-specific marker genes. Values are means ± SEM of three independent experiments. ^∆ ∆ ∆^*P* < 0.001 versus the control, and ^∗∗^*P* < 0.01, and ^∗∗∗^*P* < 0.001 versus just RANKL-treated cells.

**FIGURE 7:**
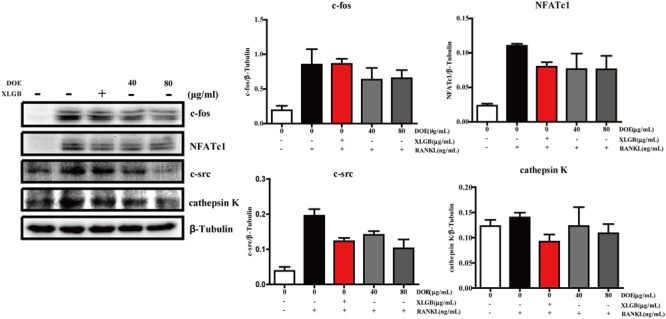
DOE treatment reduces the expression of osteoclast-specific marker proteins. In this experiment, c-Fos, NFATc1, c-Src, cathepsin K, and β-tubulin levels were all detected using western blotting, and results were quantified using the software AlphaView. Representative images are displayed; values are the means ± SEM of three independent experiments.

As our results demonstrate that DOE suppresses osteoclastogenesis, we further hypothesized that this extract would inhibit the expression of osteoclast-specific marker genes and proteins in RAW264.7 cells following RANKL stimulation. Subsequent qRT-PCR analysis (**Figure 6**) revealed that both XLGB and DOE treatments significantly inhibit the messenger RNA (mRNA) expression of osteoclast marker genes, including the nuclear factor of activated T cells (*NFATc1*), *TRAP, cathepsin K, β3-Integrin, c-Fos*, and *c-Src* (*P* < 0.01 or *P* < 0.001). In addition, consistent with these qRT-PCR results, western blot analysis also demonstrates that the protein expression of NFATc1, c-Fos, c-Src, and cathepsin K tends to be down-regulated by XLGB and DOE treatments during osteoclastogenesis (**Figure 7**). Collectively, these results strongly indicate that DOE treatment effectively inhibits RANKL-mediated osteoclastogenesis in RAW264.7 cells.

## Discussion

The orchid *D. officinale* possesses unique medicinal properties, and has been widely utilized in Asian traditional medicine for the prevention and treatment of various ailments, including obesity, diabetes, hypertension, hyperlipidemia, inflammation, and cancer (Li L. et al., 2011; Li G. et al., 2014; Wang Q. et al., 2014). A close relative, *D. moniliforme*, another medicinal orchid, has also recently been reported to exert inhibitory effects on both *in vitro* RANKL-induced osteoclast differentiation and *in vivo* lipopolysaccharide-induced bone erosion (Baek et al., 2016). Previous studies have shown, however, that the chemical composition and quantity of compounds are markedly different between these two orchids; indeed, the subject of this study, *D. officinale*, is likely of higher quality and medicinal value compared to *D. moniliforme* given the same chemical compositions (Chen et al., 2006). We therefore hypothesize that *D. officinale* can play an important role in treating osteoporosis and inhibiting osteoclast differentiation. Consistent with this hypothesis, we have shown that DOE treatment significantly mitigates osteoporosis *in vivo* and inhibits *in vitro* osteoclast differentiation. In order to carry out the experiment better, we preformed the quality assessment by chemical composition analysis. Interestingly, based on the previously study, narigenin had the effect of anti-bone resorption (Lv et al., 2013), and it was possible that narigenin played a role in anti-PMOP. The aim of our future work will be to further investigate the bioactive compounds responsible for this anti-osteoporotic effect.

As discussed, estrogen is a crucial regulator of bone quality. The role of this hormone in human bone health is well-illustrated by the fact that an estrogen deficiency can promote the development of PMOP (Miyauchi et al., 2013; Miyamoto, 2015; Chen et al., 2017; Streicher et al., 2017; Xu et al., 2017). It is also well-established that an ovariectomy can result in osteoporosis correlated with a significant increase in body weight and bone resorption, a dramatic decrease in uterine weight and BMD as well as deterioration of bone microarchitecture; all these changes are largely the result of estrogen deficiencies (Ye et al., 2015). Although hormone supplements can ameliorate these issues, as discussed above, adverse effects on other tissues are inevitable (Chen et al., 2017). Thus, the use of natural products as treatments for osteoporosis should be explored as these will have less side effects. We investigated the osteoprotective effects of DOE on PMOP in this study using an OVX rats model. Results show that DOE exhibits potent anti-osteoporotic capabilities but cannot reverse OVX-induced body weight gains. It is also noteworthy that while DOE treatment ameliorated both lipid and glucose metabolism in OVX rats at the biochemical level, these changes were not significant. Nevertheless, our results indicate that DOE treatment may inhibit osteoporosis without lowering unwanted body weight gains, while improving the metabolism of lipids and glucose.

Levels of estrogen are dramatically reduced following ovariectomies and this can seriously impair both bone mass and architecture (Khosla et al., 2012; Klein-Nulend et al., 2015). The results of this study show that both medium- and high-dose DOE treatments significantly elevated the E_2_ level in OVX rats, indicating that this extract may exert phytoestrogenic effects mainly via estrogen receptor α signaling (Imai, 2013; Streicher et al., 2017). In addition, losses of calcium and phosphorus are also obvious pathological features in patients with osteoporosis, and so treatments for osteoporosis that are based on these elements (e.g., bisphosphonates) are widely applied (Curtis et al., 2016). Interestingly, the results of this study show that OVX-induced calcium and phosphorus losses were both significantly alleviated by DOE treatment, which suggests that this extract might be able to effectively maintain homeostasis of these elements. We used a series of formation and resorption parameters to evaluate the mechanisms of DOE treatment on bone metabolic biomarkers; the activities of both ALP and BGP have been widely applied in this context as markers of bone formation (Ye et al., 2015). The results of this study show that DOE treatment significantly reduced the activities of both markers in OVX rats, indicating a decreasing rate of bone turnover. In addition, levels of ACP, another vital indicator of bone resorption (Liu et al., 2017), were significantly enhanced in OVX rats; interestingly, DOE treatment markedly suppressed the activity of ACP in OVX rats. Our results indicate that DOE treatment prevents OVX-induced osteoporosis mainly by exerting a two-way regulatory effect on bone metabolism.

Uterine atrophy is another commonly observed phenomenon in OVX rats, following a remarkable reduction in the weight of this organ (Liu et al., 2017). Notably, the results of this study show that DOE treatment significantly increased uterine weight, the organ coefficient of the uterus, and endometrial height in OVX rats, indicating that this extract can effectively reverse uterine atrophy. However, the same effect on femoral physical parameters was not seen; wet and dry weight, organ coefficients, diameter, and length all remained unaffected in OVX rats following DOE treatment. It is well-known that femoral BMD, biomechanical properties, and microarchitecture can be used as proxies for bone quality (Liu et al., 2017). The results of this study show that DOE treatment significantly increases femoral BMD in OVX rats but does not significantly change femoral biomechanical properties (i.e., maximum deflection and load). More interestingly, DOE treatment notably increases cortical bone thickness and trabecular area in the femur of OVX rats; this result strongly suggests that DOE can effectively mitigate bone microarchitectural deterioration. These results therefore suggest that DOE treatment will have a very positive protective effect on bone quality.

Bone mass is tightly regulated by a delicate balance between osteoblast formation and osteoclast resorption; the excess differentiation and activation of osteoclasts causes osteoporosis (Miyauchi et al., 2013). Thus, in order to investigate whether DOE treatment can inhibit osteoclastogenesis, we carried out TRAP staining using a series of RAW264.7 cells, one well-established preosteoclast cell line for the study of this process (Liu et al., 2017). Our results show that DOE treatment can effectively inhibit RANKL-mediated osteoclastogenesis in RAW264.7 cells and suppress the expression of osteoclast-specific marker genes and proteins during this process. Most importantly, our *in vivo* experiments show that DOE treatment exerts two-way regulatory effects on bone metabolism. Further work is needed, however, to address the question of whether, or not, DOE treatment can simultaneously inhibit bone resorption and promote formation. According to previous study on osteoclastogenesis, the signal transduction pathways including the proteins p38, c-jun N-terminal kinase (JNK), and Akt are activated in response to the direct binding of RANK and RANKL (Li et al., 2002; Ikeda et al., 2008; Moon et al., 2012). However, it currently remains unclear whether, or not, RANKL-induced phosphorylation of these signaling pathways was associated with the anti-osteoclastogenic effect of DOE. The precise mechanisms and bioactive compounds involved in osteoclastogenesis inhibition via DOE treatment need to be clarified.

## Conclusion

In this study, we have shown that DOE treatment can prevent ovariectomy-induced osteoporosis *in vivo* and inhibit RANKL-induced osteoclast differentiation *in vitro*. Our results suggest that DOE may have a promising role to play in the future treatment of osteoporosis caused by estrogen deficiencies.

## Author Contributions

JS, X-JW, and X-QF conceived and designed the experiments. QW, C-TZ, JW, Y-NW, and Y-WH performed the experiments. QW, C-TZ, and JW analyzed the data. JS and X-JW contributed reagents/materials/analysis tools. QW, C-TZ, and X-JW wrote the manuscript. All authors read and approved the final manuscript.

## Conflict of Interest Statement

The authors declare that the research was conducted in the absence of any commercial or financial relationships that could be construed as a potential conflict of interest.
