# Corrigendum: *Dendrobium officinale* Orchid Extract Prevents Ovariectomy-Induced Osteoporosis *In Vivo* and Inhibits RANKL-Induced Osteoclast Differentiation *In Vitro*

**DOI:** 10.3389/fphar.2020.00578

**Published:** 2020-04-30

**Authors:** Qi Wang, Cheng-Ting Zi, Jing Wang, Yu-Na Wang, Ye-Wei Huang, Xue-Qi Fu, Xuan-Jun Wang, Jun Sheng

**Affiliations:** ^1^Key Laboratory of Pu-erh Tea Science, Ministry of Education, Yunnan Agricultural University, Kunming, China; ^2^Tea Research Center of Yunnan, Kunming, China; ^3^College of Tea Science, Yunnan Agricultural University, Kunming, China; ^4^College of Food Science and Technology, Yunnan Agricultural University, Kunming, China; ^5^College of Life Sciences, Jilin University, Changchun, China; ^6^State Key Laboratory for Conservation and Utilization of Bio-Resources in Yunnan, Kunming, China

**Keywords:** DOE, postmenopausal osteoporosis, ovariectomy, bone quality, osteoclastogenesis

In the original article, there was a mistake in **Figure 4** as published. Panels 2 and 3 (cortical bone tissue stained with H&E for OVX Model and XLGB treated group, respectively) of **Figure 4D** in the original article was the same images as panels 2 and 3 of Figure 2E in *Liang Q, Lv M, Zhang X, Hu J, Wu Y, Huang Y, Wang X and Sheng J (2018) Effect of Black Tea Extract and Thearubigins on Osteoporosis in Rats and Osteoclast Formation in vitro. Front. Physiol. 9:1225. doi: 10.3389/fphys.2018.01225*. Based on the 3R (Reduction, Replacement, and Refinement) principle of experimental animals, the authors simultaneously and systematically evaluated the pharmacological effects of *Dendrobium officinale* Orchid extract, black tea extract, and thearubigins in preventing osteoporosis using the same batch of ovariectomized (OVX) female rats as the animal model of postmenopausal osteoporosis in the animal experiment study. They collected the data and published two articles and accidentally reused the same images in them. The corrected **Figure 4** appears below.

The authors apologize for this error and state that this does not change the scientific conclusions of the article in any way. The original article has been updated.

**Figure 4:**
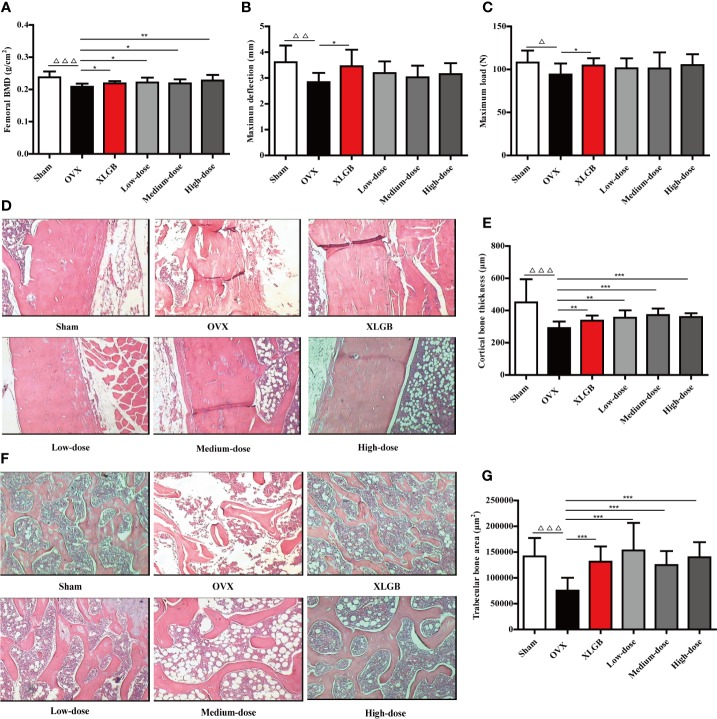
DOE treatment improves bone quality in OVX rats: **(A)** femoral BMD; **(B)** maximum deflection; **(C)** maximum load; **(D)** cortical bone tissue stained with H&E; **(E)** calculated cortical bone thickness; **(F)** trabecular bone tissue stained with H&E; and **(G)** calculated trabecular bone area. Representative images were acquired using a medical image analysis system at an original magnification of ×400. All data are presented as means ± SEM (n = 10). *^△^P* < 0.05, *^△△^P* < 0.01, and *^△△△^P* < 0.001 versus the sham group, and ^*^P < 0.05, ^**^P < 0.01, and ^***^P < 0.001 versus the OVX group.

